# Effects of n-3 polyunsaturated fatty acid supplementation on appetite: a systematic review and meta-analysis of controlled clinical trials

**DOI:** 10.1186/s13643-023-02430-y

**Published:** 2024-01-27

**Authors:** Bahareh Sasanfar, Fatemeh Toorang, Amin Salehi-Abarghouei

**Affiliations:** 1 Research Center for Food Hygiene and Safety, School of Public Health, Shahid Sadoughi University of Medical Sciences, Yazd, Iran; 2 Yazd Cardiovascular Research Center, Non-Communicable Diseases Research Institute, Shahid Sadoughi University of Medical Sciences, Yazd, Iran; 3 Department of Nutrition, School of Public Health, Faculty of Health, Shahid Sadoughi University of Medical Sciences, P O Box 8915173160, Yazd, Iran; 4 Cancer Research Center, Cancer Institute of Iran, Tehran University of Medical Sciences, Tehran, Iran; 5 Student Research Committee, Shahid Sadoughi University of Medical Sciences, Yazd, Iran; 6 Departments of Medical and Surgical Sciences, University of Bologna, Bologna, Italy

**Keywords:** n-3 polyunsaturated fatty acids, Appetite, Visual analog scale, Systematic review, Meta-analysis

## Abstract

**Background:**

The current studies explore the effect of omega-3 polyunsaturated fatty acids (PUFAs) on appetite.

**Objective:**

To examine the effect of omega-3 polyunsaturated fatty acids (n-3 PUFAs) on appetite using a systematic review and meta-analysis of controlled clinical trials (CTs).

**Patients and methods:**

Online databases including PubMed, Scopus, ISI Web of Science, and Google Scholar were searched up to January 2022. A random-effects model was used to compare the overall standardized mean difference in appetite scores between n-3 PUFAs supplemented and control individuals.

**Results:**

Fifteen eligible CTs with 1504 participants (872 for n-3 PUFA supplementation and 632 for placebo groups) were included in our systematic review. The meta-analysis showed no significant difference in overall appetite score between n-3 PUFAs supplemented and control groups (standardized mean difference [SMD] = 0.458, 95% confidence interval [CI] − 0.327, 1.242, *P* value = 0.25). However, the n-3 PUFA supplementation significantly increased the desire to eat (SMD = 1.07, 95% CI 0.116, 2.029, *P* = 0.02) compared to control.

**Conclusion:**

Although we found no effect of omega-3 supplementation on overall appetite score, it modestly increases the desire to eat. Further CTs evaluating the effect of PUFAs on appetite are still needed to confirm these findings.

**Supplementary Information:**

The online version contains supplementary material available at 10.1186/s13643-023-02430-y.

## Introduction

Dietary recommendations have emphasized the importance of fatty acid type rather than total dietary fat intake [1]. Many studies have explored the association between fatty acids’ chain length, degree of saturation, and position of the double bond of fatty acids consumed with cardiovascular diseases, inflammation, cancer, weight gain, and obesity [2–5]. Studies have also shown that saturated fatty acids (SFAs) are harmful to health, while beneficial health has been offered for monounsaturated (MUFAs) and polyunsaturated fatty acids (PUFAs) [6]. Omega-3 PUFAs’ sources include plant-based products (e.g., nuts, plant seeds, and their oils), seafood, or marine. High attention has been paid to the potential effect of different types of fatty acids on energy balance, weight, and appetite [7, 8].

Appetite is one of the important factors in controlling body weight which is regulated through both physiological and psychological factors [9]. Dietary fat composition could be changed through changes in the type of fatty acid intake which could affect the appetite [10]. Studies have examined appetite responses to meals enriched in different types of fatty acids and suggested that these different effects are via the physiochemical properties of fatty acids [11, 12]. However, there is little consensus on the relative role each may play in controlling food intake. A meta-analysis of controlled trials in patients with cancer cachexia showed n-3 PUFA supplementation did not improve body weight [13]. A study of 18 lean men showed no significant effect of fatty acid chain length on appetite [14]. Another study on 16 obese women reported that fatty acid composition did not differentially affect subjective appetite rating [15]. However, a study on 13 healthy Chinese men illustrated that PUFA-rich meals led to a decrease in appetite compared to MUFA-rich meals [5]. It is also proposed that n-3 PUFA supplementation might affect appetite control [16]. In particular, eicosapentaenoic (EPA) and docosahexaenoic acids (DHA) intake have been reported as appetite modulators [17]. The mechanisms by which n-3 PUFAs reduce appetite are not well understood. The effect of n-3 PUFAs on fat metabolism and plasma concentrations of the appetite-suppressing hormones might explain the effect [18–20]. Several clinical trials have been conducted to examine the effect of n-3 PUFA fatty acids on appetite [11, 21]. However, they have led to inconsistent results. For instance, a study done in Georgia University showed that a diet rich in PUFAs has a greater effect on appetite suppression than a diet rich in monounsaturated fat [22]. Also, consumption of a diet rich in PUFAs in fifteen healthy American men resulted in suppression of postprandial hunger [23]. However, a randomized cross-over study among sixteen healthy American females showed that a liquid meal rich in PUFAs made no significant difference in hunger, fullness, or desire to eat [10].

To address the current controversy on the effect of n-3 PUFA intake on appetite, we conducted a systematic review and meta-analysis of controlled clinical trials (CTs).

## Methods

The present study is reported following Preferred Reporting Items for Systematic Reviews and Meta-analyses [24].

### Search strategy

We conducted a systematic literature review search in PubMed/MEDLINE, Scopus, and ISI Web of Science (a list of WoS databases is in Supplementary Table 1) without language or any other restriction from the earliest available online indexing year to January 15, 2022. The search strategy included keywords and subject headings about n-3 polyunsaturated fatty acid (“Omega-3 Fatty Acid,” “Eicosapentaenoic Acid,” “EPA,” “DHA,” “docosahexaenoic acid,” “Omega-3,” “n-3,” “fish oil,”) and appetite (“Appetites,” “Appetite Alterations,” “satiety response,” “satiation,” “satiety,” “fullness,” “hunger,” “desire to eat,”). The full list of search terms used is in “Supplementary Table 1”. These searches were supplemented by reviewing the reference lists of trial publications.

### Eligibility criteria

Two investigators screened the title and abstract which was followed by the full-text assessment of the eligible articles (BS and FT). All published CTs were included if they met the following inclusion criteria: (1) clinical trials that examined the effects of n-3 PUFAs intake on appetite, (2) the questionnaire for assessing appetite should be valid or clear, (3) n-3 PUFAs consumed as a supplement (not food), (4) individuals consumed n-3 PUFAs were not supplemented with other micro- and macronutrients, (5) the type of received n-3 PUFAs should be specified (EPA and DHA), (6) appetite was reported as a score or side effects, (7) appetite should be assessed by using a valid questionnaire, and (8) participants’ age should be ≥ 18 years. Nonhuman studies were excluded.

### Screening process

Two independent authors (BS, FT) conducted the data extraction and evaluated the risk of bias. The possible discrepancies were solved by contacting the third author (ASA).

### Data extraction

The following information was extracted: the first author’s last name, the year of publication, geographic location, study design, sample size and attrition, participant’s gender, age, health condition, duration of intervention, intervention dose, and types of n-3 PUFAs, inclusion criteria, and mean (and standard deviation) score of visual analog scale (VAS).

### Risk of bias assessment

We assessed study quality using the Cochrane Collaboration’s tool [25] which takes random sequence generation, allocation concealment, blinding of participants and personnel, blinding of outcome assessment, incomplete outcome data, and selection outcome reporting into account. A judgment of “low risk of bias”, “high risk of bias”, or “unclear risk of bias” was made for each domain based on Cochrane collaboration’s handbook [26].

### Statistical analysis

The standardized difference in mean changes ± standard error (SE) in VAS score between participants assigned to n-3 PUFA supplementation and participants assigned to the control group. A random-effects model was used for calculating weighted mean differences (WMDs) and 95% confidence intervals (CIs). Cochran’s *Q* test was administered to test the statistical heterogeneity between studies. Also, we calculated the ratio of between-study variation to total variation (*I*^2^ statistic, range of this estimating is from 0 to 100%). *I*^2^ > 50% and *P* value < 0.05 were considered to indicate a significant heterogeneity between trials. Subgroup analyses based on health status, dose of PUFA supplementation, and risk of bias were administered to detect the source of potential heterogeneity between studies. Sensitivity analyses were conducted to determine if the individual study altered the results of meta-analyses significantly. The possibility of publication bias was assessed by visual inspection of a funnel plot of treatment effects versus their corresponding SE. The asymmetry was also statistically checked by using Egger’s test. The analyses were performed using STATA version 14.1 (Stata Corp, College Station, TX). *P* values < 0.05 were considered statistically significant.

## Results

### Study selection

**Fig. 1:**
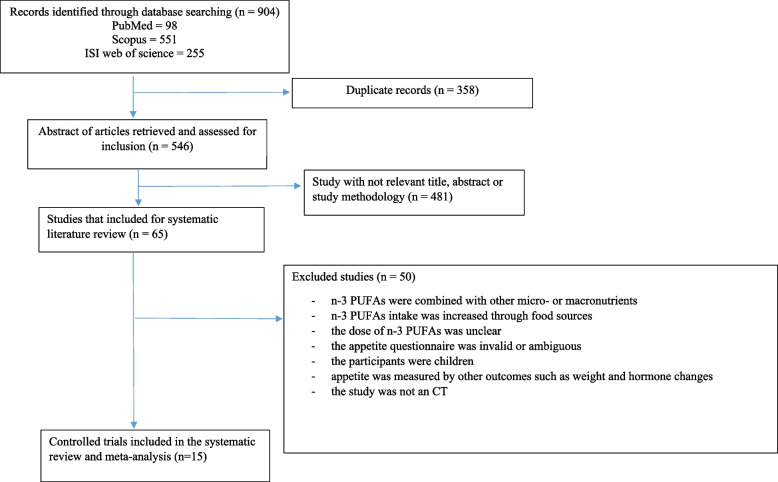
Flow diagram of study screening

A total of 553 publications were retrieved after duplicates had been removed. After reading the titles and abstracts 481 studies were excluded. We excluded 50 studies for the following reasons: n-3 PUFAs were combined with other micro- or macronutrients (*n* = 12) [27–38], n-3 PUFA intake was increased through food sources (*n* = 10) [15, 23, 39–46], the dose of n-3 PUFAs was unclear (*n* = 2) [36, 47], the appetite questionnaire was invalid or ambiguous (*n* = 1) [48], the participants were children (*n* = 7) [49–55], appetite was measured by other outcomes such as weight and hormone changes (*n* = 16) [7, 37, 56–69], or the study was not a control trial (*n* = 2) [70, 71]. Finally, 15 studies were included in the systematic review and meta-analysis (Fig. 1) [47, 52, 64, 72–83].

### Study and participants’ characteristics

Characteristics of the 15 included trials are shown in Table 1. 872 participants for n-3 PUFA supplementation and 632 for placebo in our systematic review. The sample size varied from 20 to 421 with an age range from 18 to 90 years. All included CT studies were published between 2003 to 2021. Three studies were conducted in Iran [78, 81, 82], two in Canada [72, 73], two in the USA [64, 77], and two in Israel [47, 52], and the others were conducted in Turkey [76], Denmark [83], Sweden [75], China [74], Brazil [79], and Germany [80]. Of these trials, two studies were cross-over clinical trials and the rest were parallel. The majority of them included both genders, and only two studies were conducted on male adults. The duration of n-3 PUFA supplementation ranged from two to 15 weeks. The dose of n-3 PUFAs ranged from 225 to 4.5 g/d. Six studies reported changes in appetite with the VAS questionnaire [72, 76, 78, 81–83], three studies used another valid questionnaire [47, 73, 75], and the rest reported appetite as a side effect of n-3 PUFA supplementation [52, 64, 77, 79, 80].

**Table 1:** Characteristics of studies included in the systematic review

Source (first author, year of publication)	Country	Number, sex (F/M)	Age (year)	CT design	Duration	Health status	Intervention group	Control group	Reported appetite data
Moradi et al., 2021 [82]	Iran	72 M	20–30, Int & con:22.2	Two arms Parallel	3 wk	Young male athletes with normal body fat percentage	Two omega-3 soft gel capsules/day (2000 mg omega-3; EPA:360 mg, DHA:240 mg)	Two soft gel capsules/day (1 g of edible paraffin oil)	VAS score
Safaeiyan et al., 2018 [81]	Iran	66 (22M/44F)	18–45, Int:34.2 Con:33.5	Two arms Parallel	4 wk	BMI > 30 kg/m^2^	1000 mg Omega-3 capsules twice a day (180 mg EPA & 120 mg DHA)	Paraffin soft gels twice a day	VAS score
Mocelin et al., 2017 [79]	Brazil	45 (25M/20F)	18–70, Int:56 Con:51	Two arms Parallel	9 wk	Gastrointestinal cancer patients	Two capsules of fish oil/d (3.6 g), each capsule contained 1 g EPA + 0.5 g DHA/d	Two capsules of extra virgin olive oil/d	Adverse event
Payahoo et al., 2017 [78]	Iran	60 (15M/45F)	18–45, Int:31.9 Con:33.5	Two arms Parallel	4 wk	Obese (BMI = 30–40 kg/m^2^)	1 g Omega-3 capsules twice a day (180 mg EPA & 120 mg DHA)	Placebo twice a day	VAS score
Werner et al., 2017 [80]	Germany	33 (16M/17F)	> 21 years, Int:70.3 Con:71.3	Two arms Parallel	6 wk	Pancreatic cancer	500 mg soft gel capsules 3 times/d, 60% fish oil & 40% MCT (6.9g/100g EPA + 13.6 g/100g DHA) [0.3 g n-3 fatty acids/d]	Marine phospholipids (MPL), 35% n-3 fatty acid phospholipids + 65% neutral lipids (8.5g/100g EPA + 12.3g/100g DHA)	Adverse event
Berg et al., 2014 [64]	USA	267 (184M/83F)	21–79, Int:44.2 Con:43.5	Five arms Parallel	12 wk	Adults with borderline high or high triglyceride levels	One, two, four or eight 500 mg Krill oil capsules/d (100, 200, 400 or 800 mg EPA + DHA)	Placebo (olive oil)	Adverse event
Damsbo-Svendsen, et al., 2013 [83]	Denmark	20 (10M/10F)	18–30, Int & con: 24	Cross-over	3 wk	Healthy students (> 18 y, normal weight)	3.5 g n-3 PUFAs (1.9 g EPA & 1.1 g DHA)	5.2 g soybean oil & 10 IU/g vitamin E	VAS score
Kanat et al., 2013 [76]	Turkey	62 (48M/14F)	22–84, Int:60.7	Three arms Parallel	12 wk	Cancer patients (aged ≥ 18 y)	Megestrol acetate (MA) + Meloxicam + EPA (2.2 g/d)	Megestrol acetate (MA) + Meloxicam	VAS score
Miller et al., 2013 [77]	USA	29 (17M/12F)	> 21 years, 67.4	Two-period cross-over	Two × 6 wk (2 wk washout)	Adults Diabetes patients with kidney injury	4 Capsules/d, each 1 g capsules contained PUFAs (85% n-3 [DHA:EPA ratio of 2:1])	Placebo (corn oil)	Adverse event
Vakhapova et al., 2011 [52]	Israel	131 (66Int/65Con)	50–90, Int:72.4 Con:72.7	Two arms Parallel	15 wk	Elderly	3 capsules of phosphatidylserine DHA (PS-DHA; 300 mg PS & 79 mg DHA + EPA [DHA:EPA ratio of 3:1]) /d	Placebo (cellulose)	Adverse event
Irving et al., 2009 [75]	Sweden	174 (84M/90F)	Int:73 Con:73	Two arms Parallel	24 wk	Patients with mild to moderate Alzheimer	Four 1-g capsules daily (430mg DHA & 150mg EPA)	Four 1-g corn oil (0.6g linoleic acid)	Neuropsychiatric Inventory (NPI)
Liu et al., 2007 [74]	Chinese	22 (13M/9F)	45–75, Int:56 Con:58	Two arms Parallel with 1 wk for rest	7 wk	Gastric cancer cachexia patients	8 gelatin capsules of fish oil (EPA + DHA 315mg) twice a day	Atracylenolide 6 ml (Atracylenolide Ι 0.11gml^−1)^ twice a day	VAS score
Yehuda et al., 2005 [47]	Israel	126 M	-	Two arms Parallel	3 wk	Undergraduate college students with anxiety	225 mg α-linolenic acid & linoleic acid (in ratio of 1:4) for twice daily	placebo (mineral oil)	
Jatoi et al., 2004 [73]	Canada	421 (294M/127F)	18 > , Int:66 Con:66	Three arms Parallel	4 wk	Patients aged > 18 with incurable malignancies	An EPA supplement (1.09 g EPA & 0.46 DHA), two cans/d	Megestrol acetate (MA)	NCCTG questionnaire for appetite
Bruera et al., 2003 [72]	Canada	60 (17M/43F)	Int:63.0 Con:64.6	Two arms Parallel	2 wk	Patients with advanced cancer	18 gelatin capsules containing 1000 mg fish oil (180 mg EPA & 120 mg DHA)	18 gelatin capsules containing 1000 mg of a placebo (olive oil)	VAS score

*F* female, *M* male, *Int* intervention, *Con* control, *wk* week, *CT* control trial, *PUFAs* polyunsaturated fatty acids, *DHA* docosahexaenoic acid, *EPA* eicosapentaenoic acid, *VAS* visual analogue scales, *BMI* body mass index

### Assessment of risk of bias

Of 15 studies, three were determined to have a low risk of bias [79, 82, 83], and the others were evaluated as having a high risk of bias (Table 2). All of the mentioned studies reported random sequence generation, incomplete outcome data, and selective outcome reporting as low risk of bias. However, a study done by Yehuda et al. did not report these domains. Four trials reported the method of allocation concealment [79, 80, 82, 83]. Therefore, the remaining studies were regarded as high or unclear risk of bias.

**Table 2:** Study quality and risk of bias assessment of included studies according to the Cochrane Collaboration’s tool

Fist author (year)	Random Sequence Generation	Allocation concealment	Blinding of participants and personnel	Blinding of outcome assessment	Incomplete outcome data	Selective outcome reporting	Overall quality^a^
Moradi (2021) [82]	L	L	L	L	L	L	L
Safaeiyan (2018) [81]	L	U	L	L	L	L	U
Mocellin (2017) [79]	L	L	L	L	L	L	L
Payahoo (2017) [78]	L	U	L	L	L	L	U
Werner (2017) [80]	L	L	L	U	L	L	U
Berge (2014) [64]	L	U	L	U	L	L	U
Damsbo-Svendsen (2013) [83]	L	L	L	L	L	L	L
Kanat (2013) [76]	U	U	U	U	L	L	U
Miller (2013) [77]	L	U	L	L	L	L	U
Vakhapova (2011) [52]	L	U	L	U	L	L	U
Irving (2009) [75]	L	U	U	U	L	L	U
Liu (2007) [74]	L	H	H	H	L	L	H
Yehuda (2005) [47]	H	H	H	H	H	H	H
Jatoi (2004) [73]	L	U	L	U	L	L	U
Bruera (2003) [72]	U	U	L	U	L	L	U

*U* unclear risk of bias, *L* low risk of bias, *H* high risk of bias

^a^Low quality: all criteria met; unknown quality: one criterion not met (i.e., high risk of bias for one domain or one criteria unclear); Poor quality: two or more criteria listed as high or unclear risk of bias

### Meta-analysis

Eight studies with a total of 636 participants reported data on the effect of n-3 PUFA intake on appetite [47, 72, 75, 76, 78, 81–83]. The meta-analysis showed no significant effect of n-3 PUFA intake and total appetite score (SMD = 0.458, 95% CI − 0.327, 1.242, *P* = 0.25). There was significant heterogeneity among these studies (*Q* statistic = 140.49, *P* = 0.0, *I*^*2*^ = 95.0%). The domains of VAS score including hunger, satiety, and desire to eat were reported in 4 studies. The n-3 PUFA supplementation modestly increased the desire to eat (SMD = 1.07, 95% CI 0.116, 2.029, *P* = 0.02), and the heterogeneity among these studies was significant (*Q* statistic = 32.21, *P* < 0.001,* I*^*2*^ = 91.0%). However, the changes in hunger (SMD = 1.007, 95% CI − 0.139, 2.153, *P* = 0.08) and satiety (SMD = 0.983, 95% CI − 0.597, 2.564, *P* = 0.22) were not significant. A significant heterogeneity was observed for both hunger (*I*^*2*^ = 93.7%) and satiety (*I*^*2*^ = 96.4%). Subgroup analysis was conducted based on the health status of the studies’ participants [47, 72, 75, 76, 81–84], the dose of n-3 PUFA intervention [72, 75, 76, 81–84] and studies’ risk of [47, 72, 75, 76, 81–84]. However, no significant effect on appetite was observed in any subgroup (Table 3).

**Table 3:** Meta-analysis showing the effects of n-3 PUFA supplementation on appetite in overall analysis as well subgroup analysis (all analyses were conducted using a random effect model)

Variables	No. of studies	**Meta-analysis**		**Heterogeneity**			
		WMD (95% CI)	*P* effect	*Q* statistic	*P* within group	*I*^2^ (%)	*P* between group
**Total visual analog score**							
All studies	8	0.458 (− 0.327, 1.242)	0.253	140.49	< 0.001	95	
Health status							< 0.001
Healthy	2	1.522 (− 1.013, 4.057)	0.23	31.18	< 0.001	96.8	
Obese	2	− 0.547 (− 2.11, 1.015)	0.493	16.93	< 0.001	94.1	
Cancer	2	− 0.137 (− 0.522,0.248)	0.486	0.29	0.589	0.0	
Anxiety & Alzheimer	2	1.021 (− 0.640, 2.681)	0.228	38.18	< 0.001	97.4	
Dose of intervention							
1000–3000 mg/d	5	0.33 (− 0.92, 1.58)	0.60	91.47	< 0.001	95.6	< 0.001
≥ 4000 mg/d	2	0.12 (− 0.13, 0.38)	0.34	0.58	0.44	0.0	
Risk of bias							< 0.001
Low risk	2	1.522 (− 1.013, 4.057)	0.239	31.18	< 0.001	96.8	
Unknown	5	− 0.22 (− 0.75, 0.30)	0.40	24.33	< 0.001	83.6	
High risk	1	**1.87 (1.42, 2.32)**	** < 0.001**	51.36	< 0.001	94.2	
**Hunger score**	4	1.007 (− 0.139, 2.153)	0.08	47.31	< 0.001	93.7	
**Satiety score**	4	0.983 (− 0.597, 2.564)	0.22	83.68	< 0.001	96.4	
**Desire to eat score**	4	**1.073 (0.116, 2.029)**	**0.02**	33.21	< 0.001	91.0	

*WMD* weighted mean difference

### Sensitivity analysis and publication bias

**Fig. 2:**
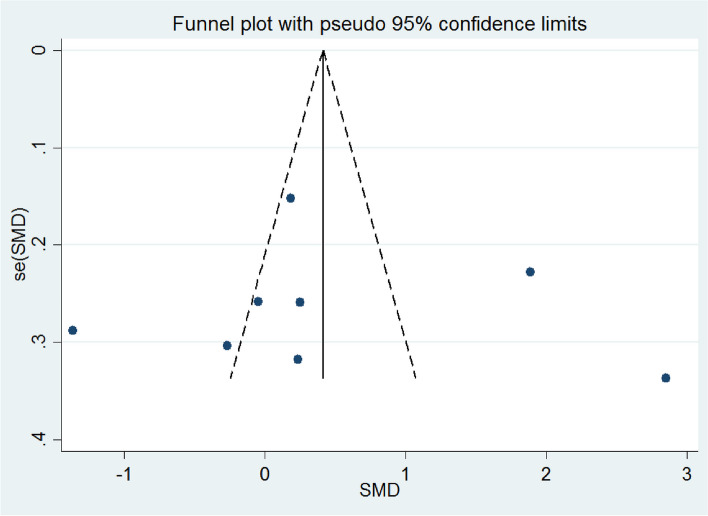
Funnel plots (with pseudo 95% CI) depicting the effect sizes (difference in means) versus their standard errors (SEs) for controlled trials that assessed the effect of n-3 PUFA supplementation on appetite

In the sensitivity analysis, none of the included studies significantly influenced the pooled effects. The final result of the sensitivity analysis was shown in Table 4. Visual inspection of the funnel plot (Fig. 2) and Egger’s test (slope = 0.067; CI − 3.82–3.95, intercept value = 0.442) showed no significant publication bias.

**Table 4:** Result of sensitivity analysis

Study omitted	Estimate	95% confidence interval	
Ozkan Kanat, 2013 [76]	1.2033992	− .50218731	2.9089859
By Eduardo Bruera, 2003 [72]	1.2033992	− .50218731	2.9089859
Abdolrasoul Safaeiyan, 2018 [81]	1.4308839	− .32518974	3.1869574
L.Payahoo, 2017 [78]	1.2033992	− .50218731	2.9089859
Signe Damsbo-Svendsen, 2013 [83]	1.2459544	− .47955447	2.9714632
Shlomo Yehuda, 2005 [47]	1.7502558	− .02857373	3.5290854
Gerd Faxen Irving, 2009 [75]	1.2033992	− .50218731	2.9089859
Sara Moradi, 2021 [82]	1.2033992	− .50218731	2.9089859
Combined	1.2033992	− .50218731	2.9089858

## Discussion

The present study demonstrated that n-3 PUFAs fatty acid supplementation had no significant effect on overall appetite. However, it modestly increases the desire to eat in adults. To the best of our knowledge, no systematic review and meta-analysis has been published in this regard.

Previous meta-analyses have investigated the relationship between n-3 PUFA intake as supplements or in the context of foods. Furthermore, some studies assessed body weight, appetite hormones, or their gene expressions in humans as markers for appetite. A review by Behroz et al. showed that polyunsaturated fats, such as n-3 and n-6, have a similar effect on increasing energy expenditure, but they differ in how they regulate weight and appetite [85]. A meta-analysis of fifty-two trials illustrated that more than 2000 mg n-3 PUFA intake for more than 10 weeks significantly increased plasma adiponectin levels, but had no significant effect on circulating leptin levels [86]. In a meta-analysis of 22 studies, it is also revealed that omega-3 polyunsaturated fatty acid PUFA supplementation in patients with cancer significantly increases body weight and plasma total ω-3 fatty acids [87]. However, a meta-analysis by Satogami et al. showed that patients with eating disorders had higher levels of n-3 PUFAs in peripheral blood tissues than in controls [88]. In contrast, a recent review on the relationship between dietary fatty acids and appetite reported that an increase in n-3 PUFAs led to higher levels of plasma appetite-suppressing hormones and satiety sensation [85]. However, our study did not find any evidence for this effect.

In our study, n-3 PUFAs fatty acid supplementation significantly increases the desire to eat in adults. However, in a study using sunflower and flaxseed oil as a high-fat diet, PUFA did not find a statistically significant effect on the desire to eat among normal-weight females [10]. The small number of studies included in the meta-analysis might have influenced the effect we observed.

Appetite is controlled via multiple physiologic processes. The mechanism by which PUFAs might change the appetite has not yet been completely explicated. However, several mechanisms were proposed for n-3 PUFA’s effects on appetite. Intracellular long-chain fatty acids of the hypothalamus may increase by n-3 PUFA intake which results in initiating satiety signals and regulating appetite [89]. Also, n-3 PUFAs activate free fatty acid receptor 4, which results in increased intracellular calcium concentration that leads to the secretion of hormones like NPY, which can decrease appetite. Some studies also found that n-3 PUFA supplements stimulate the release of bile acid and cholecystokinin which reduce the appetite [82]. However, not all individuals need to reduce their appetite. For example, patients with cancer might have poor appetite due to cytokine inhibition of neuropeptide Y. On the other hand, supplementation with n-3 PUFAs can decrease the production of interleukin-1 and interleukin-6 cytokines, then may combat the loss of appetite in these patients [90]. Therefore, n-3 PUFAs may play a role in regulating total energy intake, managing both over and under-intake [7, 87].

The current study has several limitations that should be considered. First, the included studies were conducted on participants with different conditions like healthy adults, patients with cancer, and obesity and had different intervention periods. Furthermore, a limited number of studies assessed appetite by using subjective tools. Also, none of the 8 studies in our meta-analysis evaluated the daily omega-3 intake of participants via foods. Therefore, supplementation with n-3 PUFAs may meet daily requirement intake (DRI) and the individual may not have consumed more than DRI. Also, in the included studies the dose of n-3 PUFAs for intervention ranged from 225 to 4.5 g/d. Based on the risk of bias assessment, most of the included studies were judged to be “unclear” regarding their risk of bias. Moreover, only one study belonged to the high-risk group in the subgroup analysis of risk of bias, which might limit the power of subgroup analyses in meta-analyses. Therefore, the results should be treated with caution. Finally, we did not perform the search in Cochrane, so a small number of articles may not have been included in the search results, but we compensated for this by extensive searching in other databases and referencing the included articles.

## Conclusion

The findings of the present systematic review and meta-analysis showed that the n-3 PUFA supplementation has no significant effect on appetite; however, it might increase the desire to eat. Regarding the different effects of n-3 PUFAs in healthy and unhealthy subjects with different diseases, more trials that investigate these different outcomes are needed.

### Supplementary Information

**Additional file 1: Table S1.** The search strategy used to search different databases.

**Additional file 2: Table S2.** Dose of DHA for intervention (studies used for Meta-analysis).

## Data Availability

The data underlying this article was provided in the Supplementary Table 2.
