# Supplementary material for: Effects of n-3 polyunsaturated fatty acid supplementation on appetite: a systematic review and meta-analysis of controlled clinical trials
Source: Syst Rev. 2024 Jan 27;13:44. doi: 10.1186/s13643-023-02430-y (PMC10821539; doi:10.1186/s13643-023-02430-y)
Supplement: Supplementary file 1 — Additional file 1: Table S1. The search strategy used to search different databases. [file 13643_2023_2430_MOESM1_ESM.docx]

Table of the search strategy

Supplementary Table 1- The search strategy used to search different databases.

| database | Search strategy |
| --- | --- |
| PubMed = 98 | ("Fatty Acids, Omega-3"[Mesh] OR "Eicosapentaenoic Acid"[Mesh] OR "Omega-3 Fatty Acid" [tiab] OR "Eicosapentaenoic Acid " [tiab] OR "Omega-3 fatty acid"[tiab] OR "Omega-3 fatty acids"[tiab] OR "n-3 oil" [tiab] OR "n 3 oil"[tiab] OR "n3 oil"[tiab] OR "n-3 Fatty Acids" [tiab] OR "Omega 3 Fatty Acids"[tiab] OR "n-3 PUFA"[tiab] OR "n3 Fatty Acid"[tiab] OR "Fatty Acid, n3"[tiab] OR "n3 PUFA"[tiab] OR "n3 Polyunsaturated Fatty Acid"[tiab] OR "n3 Oils"[tiab] OR "n-3 Oils "[tiab] OR "n 3 Oils"[tiab] OR "N-3 Fatty Acid"[tiab] OR "Acid, N-3 Fatty "[tiab] OR "Fatty Acid, N-3"[tiab] OR "N 3 Fatty Acid"[tiab] OR "n-3 Polyunsaturated Fatty Acid"[tiab] OR "n 3 Polyunsaturated Fatty Acid"[tiab]) AND ("Appetite"[Mesh] OR "Appetites" [tiab] OR "Appetite Alterations"[MeSH] OR "Appetite Alteration" [tiab] OR "satiety response"[MeSH] OR "satiation" [MeSH] OR "satiety"[tiab] OR "satiation"[tiab] OR "fullness"[title]) |
| Scopus = 551 | (TITLE-ABS-KEY ("Fatty Acids Omega-3") OR TITLE-ABS-KEY ("Eicosapentaenoic Acid") OR TITLE-ABS-KEY ("Omega-3 Fatty Acid") OR TITLE-ABS-KEY ("Eicosapentaenoic Acid") OR TITLE-ABS-KEY ("Omega-3 fatty acids") OR TITLE-ABS-KEY ("n-3 oil") OR TITLE-ABS-KEY ("n 3 oil ") OR TITLE-ABS-KEY ("n-3 Fatty Acids") OR TITLE-ABS-KEY ("Omega 3 Fatty Acids") OR TITLE-ABS-KEY ("n-3 PUFA") OR TITLE-ABS-KEY ("n3 Fatty Acid") OR TITLE-ABS-KEY ("n3 PUFA") OR TITLE-ABS-KEY ("PUFA, n3") OR TITLE-ABS-KEY ("n3 Polyunsaturated Fatty Acid") OR TITLE-ABS-KEY ("n3 Oils") OR TITLE-ABS-KEY ("n-3 Oils ") OR TITLE-ABS-KEY ("N-3 Fatty Acid") OR TITLE-ABS-KEY ("N 3 Fatty Acid") OR TITLE-ABS-KEY ("n-3 Polyunsaturated Fatty Acid") OR TITLE-ABS-KEY ("n 3 Polyunsaturated Fatty Acid")) AND (TITLE-ABS-KEY ("Appetite") OR TITLE-ABS-KEY ("Appetites") OR TITLE-ABS-KEY ("Appetite Alterations") OR TITLE-ABS-KEY ("Appetite Alteration") OR TITLE-ABS-KEY ("satiety response") OR TITLE-ABS-KEY ("satiation") OR TITLE-ABS-KEY ("satiety") OR TITLE-ABS-KEY ("satiation") OR TITLE-ABS-KEY ("fullness")) |
| ISI Web of Science = 255 | (TS= ("Fatty Acids Omega-3") OR TS= ("Eiosapentaenoic Acid") OR TS= ("Omega-3 Fatty Acid") OR TS= ("Eicosapentaenoic Acid ") OR TS= ("Omega-3 fatty acids") OR TS= ("Omega-3 fatty acids") OR TS= ("n-3 oil") OR TS= ("n 3 oil") OR TS= ("n-3 Fatty Acids") OR TS= ("Omega 3 Fatty Acids") OR TS= ("n-3 PUFA") OR TS= ("n 3 PUFA") OR TS= ("n3 Fatty Acid") OR TS= ("Fatty Acid n3") OR TS= ("n3 PUFA") OR TS= ("PUFA n3") OR TS= ("n3 Polyunsaturated Fatty Acid") OR TS= ("n3 Oils") OR TS= ("n-3 Oils") OR TS= ("n 3 Oils") OR TS= ("N-3 Fatty Acid") OR TS= ("Fatty Acid N-3") OR TS= ("N 3 Fatty Acid") OR TS= ("n-3 Polyunsaturated Fatty Acid") OR TS= ("n 3 Polyunsaturated Fatty Acid")) AND (TS= ("Appetite") OR TS= ("Appetites") OR TS= ("Appetite Alterations") OR TS= ("Appetite Alteration") OR TS= ("satiety response") OR TS= ("satiation") OR TS= ("satiety") OR TS= ("satiation") OR TS= ("fullness")) |

**List of WoS databases:**

Web of Science Core Collection (1900-present)

Current Contents Connect (1998-present)

Derwent Innovations Index (1980-2009)

KCI-Korean Journal Database (1980-present)

MEDLINE® (1950-present)

ProQuest™ Dissertations & Theses Citation Index

SciELO Citation Index (2002-present)

Zoological Record (2010-present)
